# Angiogenic inhibitor pre‐administration improves the therapeutic effects of immunotherapy

**DOI:** 10.1002/cam4.5696

**Published:** 2023-02-19

**Authors:** Mineyoshi Sato, Nako Maishi, Yasuhiro Hida, Aya Yanagawa‐Matsuda, Mohammad Towfik Alam, Jun Sakakibara‐Konishi, Jin‐Min Nam, Yasuhito Onodera, Satoshi Konno, Kyoko Hida

**Affiliations:** ^1^ Vascular Biology and Molecular Pathology Faculty of Dental Medicine and Graduate School of Dental Medicine, Hokkaido University Sapporo Japan; ^2^ Department of Respiratory Medicine, Faculty of Medicine Hokkaido University Sapporo Japan; ^3^ Department of Cardiovascular and Thoracic Surgery, Faculty of Medicine Hokkaido University Sapporo Japan; ^4^ Advanced Robotic and Endoscopic Surgery, School of Medicine Fujita Health University Toyoake Japan; ^5^ Global Center for Biomedical Science and Engineering (GCB), Faculty of Medicine Hokkaido University Sapporo Japan

**Keywords:** angiogenesis inhibitors, immune checkpoint inhibitors, immunotherapy, lung neoplasms, tumor microenvironment

## Abstract

In lung cancer, immune checkpoint inhibitors (ICIs) are often inadequate for tumor growth inhibition. Angiogenic inhibitors (AIs) are required to normalize tumor vasculature for improved immune cell infiltration. However, in clinical practice, ICIs and cytotoxic antineoplastic agents are simultaneously administered with an AI when tumor vessels are abnormal. Therefore, we examined the effects of pre‐administering an AI for lung cancer immunotherapy in a mouse lung cancer model. Using DC101, an anti‐vascular endothelial growth factor receptor 2 (VEGFR2) monoclonal antibody, a murine subcutaneous Lewis lung cancer (LLC) model was used to determine the timing of vascular normalization. Microvessel density (MVD), pericyte coverage, tissue hypoxia, and CD8‐positive cell infiltration were analyzed. The effects of an ICI and paclitaxel after DC101 pre‐administration were investigated. On Day 3, increased pericyte coverage and alleviated tumor hypoxia represented the highest vascular normalization. CD8+ T‐cell infiltration was also highest on Day 3. When combined with an ICI, DC101 pre‐administration significantly reduced PD‐L1 expression. When combined with an ICI and paclitaxel, only DC101 pre‐administration significantly inhibited tumor growth, but simultaneous administration did not. AI pre‐administration, and not simultaneous administration, may increase the therapeutic effects of ICIs due to improved immune cell infiltration.

## Graphical abstract

A pre‐angiogenesis inhibitor strategy improves immune checkpoint inhibitor effects by improving CD8 infiltration and drug delivery.The current clinical protocol of administering AI and ICI on the same day results in ICI acting when vascular normalization is insufficient to mobilize CTL in the tumor tissue. In contrast, the anti‐tumor effect is enhanced when AI is administered several days before ICI, as ICI is given after vascular normalization and the number of immune cells infiltrating into the tissue has increased.

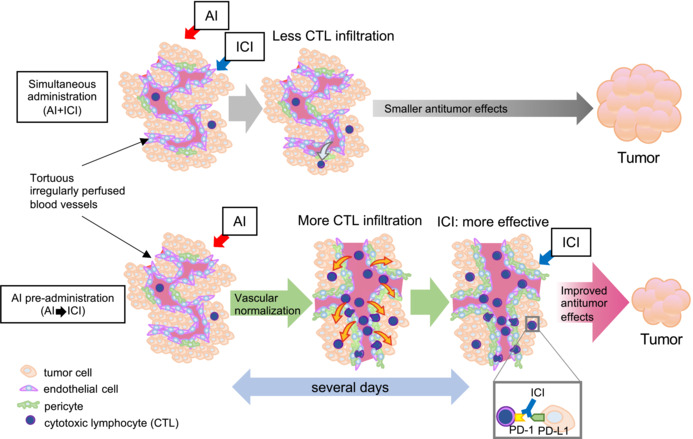

## 1 INTRODUCTION

Lung cancer has been known for its poor prognosis and is one of the most common cancer types.^1^ Advanced stage non‐small cell lung cancer harboring oncogenic driver mutations such as epidermal growth factor receptor (EGFR) gene mutations, anaplastic lymphoma kinase (ALK) gene rearrangements, c‐Ros oncogene 1 (ROS1) gene rearrangements, and B‐rapidly accelerated fibrosarcoma (BRAF) gene mutations are treated with corresponding tyrosine kinase inhibitors.^2^ Driver mutation‐negative advanced stage lung cancer is currently treated using conventional cytotoxic drugs, angiogenic inhibitors (AIs), and immune checkpoint inhibitors (ICIs).^3^ Although several studies have proven that combination therapy using these drugs is effective, patient prognoses have remained to be poor.

AIs target tumor blood vessels,^4^, ^5^ which are morphologically and functionally abnormal.^6^, ^7^ Excessive vascular endothelial growth factor (VEGF) production by tumor cells promotes excessive angiogenesis^8^, ^9^; blood vessels are tortuous and lack normal hierarchical structures.^10^, ^11^ Moreover, adhesion between endothelial cells becomes weakened, and pericyte coverage is diminished.^12^, ^13^ This could lead to increased vascular permeability and plasma leakage,^14^, ^15^, ^16^ thereby elevating interstitial pressure.^17^, ^18^ Therefore, blood vessels are obstructed, and blood flow becomes irregular and impaired.^19^ As a consequence, tumor tissues are prone to hypoxia,^20^, ^21^ while intratumoral drug delivery and immune cell infiltration are decreased.^22^ These abnormal features of the tumor microenvironment can be improved by AIs, which are known to inhibit the disruption of endothelial cell–cell adhesion by VEGF, which induces VE‐cadherin endocytosis.^13^ In tumor cells, AIs also promote pericyte recruitment to tumor blood vessels by upregulating angiopoietin‐1,^23^ which improves vessel wall integrity and drug infiltration into tumor tissues.^24^ Therefore, AIs do not merely suppress angiogenesis; they normalize abnormal blood vessels making them less leaky and more mature and functional.

The cancer–immunity cycle is composed of different factors to elicit effective anti‐tumor immunity, but crucial components are tumor‐infiltrating lymphocytes.^25^ T‐cell trafficking and infiltration into tumor tissue require functional blood vessels^26^; therefore, the vascular normalization effects of AIs are beneficial for anti‐tumor immunity.^26^, ^27^ Preclinical studies have shown that antiangiogenic therapy promotes anti‐tumor immune responses by increasing tumor‐specific cytotoxic lymphocyte infiltration^28^, ^29^ and potentiating the effectiveness of ICIs.^30^, ^31^, ^32^ In the IMpower150 clinical trial, atezolizumab (an ICI) in combination with carboplatin, paclitaxel, and bevacizumab (an AI) was found to be more effective in improving patient survival than combination of those drugs without atezolizumab.^33^ While combination therapy prolonged median patient progression‐free survival, the difference was 1.5 months, which was not adequate and indicated other strategies were required to boost the antitumoral efficacy of ICIs.

It was proposed that AIs should be administered at the optimal timing for vascular normalizing effects, as these effects are transient.^34^, ^35^ Animal and mathematical model studies supported this theory by identifying enhanced combinational effects of AIs and cytotoxic chemotherapeutic drugs from AI pre‐administration several days prior to other drugs, rather than concomitant treatments.^36^, ^37^, ^38^ These studies demonstrated that normalized tumor vasculature facilitated intratumoral blood flow and drug delivery, resulting in augmented anti‐tumor responses. However, in clinical practice, AIs are administered on the same day as ICIs and other antineoplastic drugs, which could mean other drugs are administered before vascular normalization by AI. We hypothesized that these poor drug effects were caused by insufficient drug delivery or immune cell infiltration, as tumor blood vessels were immature and nonfunctional when ICI and other antineoplastic drugs were administered. To date, no animal model studies have been conducted to address the effects of an AI pre‐administration step for lung cancer immunotherapy. Therefore, we investigated the effects of AI pre‐administration, ahead of ICI, in an immunocompetent lung cancer mouse model.

## 2 METHODS

### 2.1 Study mice

We purchased ninety 6‐week‐old male mice (C57BL/6JJcl, CLEA Japan, Tokyo, Japan) and fifteen 6‐week‐old female mice (BALB/cAJcl, CLEA Japan) and housed animals under specific pathogen‐free conditions. All animal procedures were performed in accordance with institutional guidelines and approved by the Institutional Animal Care and Use Committee of Hokkaido University.

### 2.2 Plasmids and transfections

To detect metastatic lesions by bioluminescence imaging, Venus‐Rluc‐expressing tumor cells were generated as previously described.^39^ The Rluc cDNA was kindly provided by Y. Ohmiya (National Institute of Advanced Industrial Science and Technology, Tsukuba, Ibaraki, Japan). It was amplified by PCR and cloned into pCR‐Blunt II‐TOPO (Invitrogen, Life Technologies, Carlsbad, CA, USA). The product was then sub‐cloned into the *Xho*I and *Not*I restriction sites of the pCAGGS‐Venus plasmid,^40^ and DNA fragments encoding Venus and Rluc were then sub‐cloned into the *EcoR*I and *Not*I restriction sites of pCS II‐CMV‐MCS (provided by H. Miyoshi, RIKEN Tsukuba Institute, Tsukuba, Ibaraki, Japan). Lentiviral vectors, together with the packaging vector pCAG‐HIVgp and the VSV‐G‐ and REV‐expression construct pCMV‐VSV‐G‐RSV‐REV (H. Miyoshi), were introduced into 293 T cells (purchased from RIKEN Cell Bank (Tsukuba, Ibaraki, Japan)) using FuGENE HD Transfection Reagent (Promega, Madison, WI, USA) in accordance with manufacturer's instructions to generate lentiviral particles. Lentivirus‐mediated gene transfer into Lewis lung carcinoma (LLC) cells was performed as previously described.^41^

### 2.3 Cell culture

LLC and colon‐26 cells were purchased from RIKEN Cell Bank (Tsukuba, Ibaraki, Japan). LLC and LLC‐Venus‐Rluc cells were cultured in DMEM (Sigma‐Aldrich, St. Louis, MO, USA, #D5796), supplemented with 10% fetal bovine serum and 1% penicillin/streptomycin. Colon‐26 cells were cultured in RPMI‐1640 (Sigma‐Aldrich, #R8758) and supplemented with 10% fetal bovine serum and 1% penicillin/streptomycin. Cells were cultured at 37°C in a humidified atmosphere containing 5% CO_2_. All cells were free of mycoplasma contamination.

### 2.4 Chemicals and antibodies

The following chemicals and antibodies were used: paclitaxel (Pfizer, Tokyo, Japan), InVivoMAb anti‐mouse vascular endothelial growth factor 2 (VEGFR2) antibody (DC101, BioXCell, Lebanon, NH, USA, #BE0060), InVivoMAb anti‐mouse programmed cell death 1 (PD‐1) antibody (RMP1‐14, BioXCell, #BE0416), InVivoMab rat IgG1 isotype control (TNP6A7, BioXCell, #BE0290), InVivoMab rat IgG2a isotype control (2A3, BioXCell, #BE0089), rabbit anti‐CD31 antibody (Abcam, Cambridge, UK, #ab28364), purified rat anti‐mouse CD31 antibody (BD Pharmingen, San Diego, CA, USA, #553370), rabbit anti‐mouse/human α‐SMA antibody (Abcam, #ab5694), rat purified anti‐mouse CD8α antibody (BioLegend, San Diego, CA, USA, #100702), Alexa Fluor 647 rat anti‐mouse CD31 antibody (BioLegend, #102416), rabbit anti‐mouse glucose transporter 1 (GLUT1) antibody (Abcam, #ab115730), goat anti‐mouse programmed cell death‐ligand 1 (PD‐L1) antibody (R&D systems, Minneapolis, MN, USA, #AF1019), rabbit anti‐mouse CD8α antibody (Abcam, #ab217344), rat anti‐mouse granzyme B monoclonal antibody (Invitrogen, Carlsbad, CA, USA, #14–8822‐82), APC rat anti‐mouse/human CD11b antibody (Biolegend, #101212), FITC rat anti‐mouse Ly‐6G/Ly‐6C (Gr‐1) antibody (Biolegend, #108405), Alexa Fluor 647 goat anti‐rat IgG antibody (BioLegend, #405416), Alexa Fluor 488 goat anti‐rabbit IgG antibody (Invitrogen, #A11034), Alexa Fluor 488 goat anti‐rat IgG antibody (Invitrogen, #A11006), Alexa Fluor 546 goat anti‐rat IgG antibody (Invitrogen, #A11081), Alexa Fluor 647 goat anti‐rabbit IgG antibody (Invitrogen, #A21244), goat anti‐rabbit IgG antibody/horse radish peroxidase (HRP) (DAKO, Carpinteria, CA, USA, #P0448), and rabbit anti‐goat IgG antibody/HRP (DAKO, #P0449). We also used in vivo grade VivoGlo Luciferin (Promega, Madison, WI, USA, #P1043) and DAPI solution (Dojindo, Kumamoto, Japan, #PQ012).

### 2.5 In vivo mouse syngeneic lung cancer model

To prepare an AI monotherapy model, 1 × 10^6^ LLC cells in 100 μL of Hanks' Balanced Salt Solution (HBSS) (Gibco, Life Technologies) were subcutaneously inoculated into the right flank of 40 mice. When tumor volume exceeded 45 mm^3^, one mouse group (Group 1, *n* = 5/group) was sacrificed (Day 0), while five groups were treated with intraperitoneal anti‐VEGFR2 monoclonal antibody (DC101 at 800 μg/mouse) and sacrificed after 1, 3, 5, 7, and 14 days (Groups 2, 3, 4, 5, and 6, respectively). One group (Group 7, *n* = 5/group) was treated with a second dose of DC101 on Day 7. One group (Group 8, *n* = 5/group) was treated with the isotype control (TNP6A7 at 800 μg/mouse) on Day 0. These two groups were sacrificed on Day 14.

For the AI and ICI combination model, 1 × 10^6^ LLC‐Venus‐Rluc cells/100 μL HBSS were injected in the 30 mice's right flank. The tumor‐bearing mice were divided into six groups (*n* = 5/group). Group 1 received simultaneous intraperitoneal administration of TNP6A7 (isotype control for DC101, 800 μg/mouse), and 2A3 (isotype control for RMP1‐14, an anti‐mouse PD‐1 monoclonal antibody (ICI), 200 μg/mouse) on Day 14. Group 2 received TNP6A7 on Day 11 and 2A3 on Day 14. Group 3 received TNP6A7 and RMP1‐14 (200 μg/mouse) on Day 14. Group 4 mice were treated with simultaneous (sim) DC101 (800 μg/mouse) and RMP1‐14 on Day 14 (Sim AI). Group 5 mice were treated with TNP6A7 on Day 11 and RMP1‐14 on Day14. Group 6 mice were treated with DC101 on Day 11 and RMP1‐14 on Day 14 (AI first). All mice were sacrificed on Day 21. After harvesting the lungs from humanely euthanatized animals, lung metastasis was analyzed by ex vivo bioluminescence imaging using the IVIS Spectrum computed tomography system (PerkinElmer, Waltham, MA, USA).

In the AI, ICI, and paclitaxel (PTX) combination model, 1 × 10^6^ LLC cells in 100 μL HBSS were subcutaneously inoculated into the right flank of 20 mice. On post‐inoculation Day 11, mice were divided into four groups (*n* = 5/group): 1: control, 2: ICI + PTX, 3: sim AI, and 4: AI first groups. The control group received no treatment, the ICI + PTX group received RMP1‐14 (10 mg/kg) + PTX (20 mg/kg) on Days 14 and 21, the sim AI group received DC101 (40 mg/kg) + RMP1‐14 (10 mg/kg) + PTX (20 mg/kg) on Days 14 and 21, and the AI first group received DC101 (40 mg/kg) on Days 11 and 18 + RMP1‐14 (10 mg/kg) + PTX (20 mg/kg) on Days 14 and 21. All drugs were intraperitoneally administered. Mice were euthanized, and tissues were harvested on Day 26. Tumor volumes were calculated as 0.5 × length × width^2^.

### 2.6 In vivo mouse syngeneic colon cancer model

8 × 10^5^ colon‐26 cells in 100 μL of HBSS were subcutaneously inoculated into 15 BALB/cAJcl mice's right flank. After Day 7 of inoculation, the mice were randomly divided into three groups (*n* = 5/group): control, sim AI and AI first. The control group (Ctrl) received TNP6A7 (40 mg/kg) + 2A3 (10 mg/kg) on Days 10 and 17, the sim AI group received DC101 (40 mg/kg) + RMP1‐14 (10 mg/kg) on Days 10 and 17 and the AI first group received DC101 (40 mg/kg) on Days 7 and 14 and RMP1‐14 (10 mg/kg) on Days 10 and 17. All drugs were intraperitoneally administered. The mice were euthanized and tissues were harvested on Day 24. Tumor volumes were calculated as 0.5 × length × width^2^.

### 2.7 Histology and immunohistochemistry

Tumor tissues were dissected from mice and halved; one‐half was embedded in Tissue‐Tek OCT compound (Sakura Finetek Japan, Tokyo, Japan) and immersed in liquid nitrogen. The other half was fixed in 10% formaldehyde, dehydrated, and embedded in paraffin. Then, paraffin‐embedded and fresh frozen tumor sections were prepared. Frozen sections were fixed in 4% paraformaldehyde or methanol. For microvessel density (MVD) analysis, paraffin‐embedded sections were stained with rabbit anti‐CD31 antibody and goat HRP‐conjugated anti‐rabbit IgG. MVD was examined by selecting five hotspots of densely distributed vessels^42^ and counting all individual CD31‐positive areas. For pericyte coverage, cryosections were stained with rat anti‐mouse CD31 and rabbit anti‐mouse/human α‐SMA antibodies and visualized with Alexa Fluor 647‐conjugated goat anti‐rat IgG and Alexa Fluor 488‐conjugated goat anti‐rabbit IgG antibodies. Coverage was calculated as the ratio of α‐SMA‐positive to CD31‐positive areas.^12^

For hypoxic tumor area assessment, paraffin‐embedded sections were stained with rabbit anti‐mouse GLUT1 and goat HRP‐conjugated anti‐rabbit IgG antibodies. Whole tumor section areas were analyzed, and hypoxic tumor areas were calculated as the ratio of GLUT1‐positive areas to the whole tumor area, excluding necrotic areas. Tumor‐infiltrating lymphocytes were visualized using the rat purified anti‐mouse CD8α and Alexa Fluor 647‐conjugated goat anti‐rat IgG or rabbit anti‐mouse CD8α and goat HRP‐conjugated anti‐rabbit IgG antibodies. CD8α‐positive areas were analyzed in five randomly selected areas or in the whole tumor area, excluding necrotic areas. In the AI monotherapy model, they were also analyzed in vascular hotspots. Granzyme B and CD8‐positive lymphocytes were visualized to analyze the activation status of lymphocytes using rat anti‐mouse granzyme B antibody, rabbit anti‐mouse CD8α antibody, Alexa Fluor 546 goat anti‐rat IgG antibody, and Alexa Fluor 647 anti‐rabbit IgG antibody. The granzyme B‐positive CD8α‐positive cell ratio was calculated. Myeloid‐derived suppressor cells (MDSCs) infiltration was analyzed as CD11b and Gr‐1 positive areas in five randomly selected areas. PD‐L1‐positive areas were analyzed by staining with goat anti‐mouse PD‐L1 and rabbit HRP‐conjugated anti‐goat IgG antibodies; areas were calculated as the ratio of PD‐L1‐positive to whole tumor areas, excluding necrotic areas. Hematoxylin–eosin staining was used to analyze tumor necrotic area. All immunohistochemistry slides were counterstained with hematoxylin, and immunofluorescence slides were nuclear‐counterstained with DAPI. Histological slides were imaged using a batch slide scanner (NanoZoomer 2.0‐HT, Hamamatsu Photonics, Hamamatsu, Shizuoka, Japan) and digital viewer (NDP.view2). MVD was analyzed using Adobe Photoshop, and other immunohistochemical images were analyzed using ImageJ software from the National Institutes of Health (Bethesda, MD, USA). Fluorescent images were randomly obtained using a KEYENCE BZ‐X810 fluorescent microscope and analyzed using the BZ‐X800 analyzer (KEYENCE, Osaka, Japan).

### 2.8 RNA isolation and quantitative real‐time PCR

Total RNA was isolated using TRIzol reagent (Life Technologies) and ReliaPrep RNA Cell Miniprep System (Promega) from tumor specimens according to manufacturer's instructions. Then, cDNA was synthesized using ReverTra Ace (Toyobo, Osaka, Japan). Quantitative real‐time PCR was performed using the KAPA SYBR FAST qPCR kit (Kapa Biosystems, Wilmington, MA, USA). Cycling conditions were set according to the CFX manager (Bio‐Rad, Hercules, CA, USA). Granzyme B and interferon‐γ (IFN‐γ) mRNA expression levels in CD8‐positive T cells were normalized to CD8α expression levels using the 2^−ΔΔCt^ method. The following primers were used: CD8α, forward, 5′‐AGCCCCAGAGACCAGAAGATTG‐3′, reverse, 5′‐CATTTGCAAACACGCTTTCGGC‐3′, Granzyme B, forward, 5′‐TACTGCTGACCTTGTCTCTGGC‐3′, reverse, 5′‐TGACTTGCTGGGTCTTCTCCTG‐3′, IFN‐γ, forward, 5′‐TGGAGGAACTGGCAAAAGGATG‐3′, reverse, 5′‐CGCTTATGTTGTTGCTGATGGC‐3′.

### 2.9 Statistical analysis

Data differences were evaluated using Student's *t*‐test between two groups and one‐way ANOVA or Tukey–Kramer test between three or more groups. *p* < 0.05 was considered statistically significant.

## 3 RESULTS

### 3.1 Analysis of vascular normalization timing in LLC tumors using DC101

**FIGURE 1:**
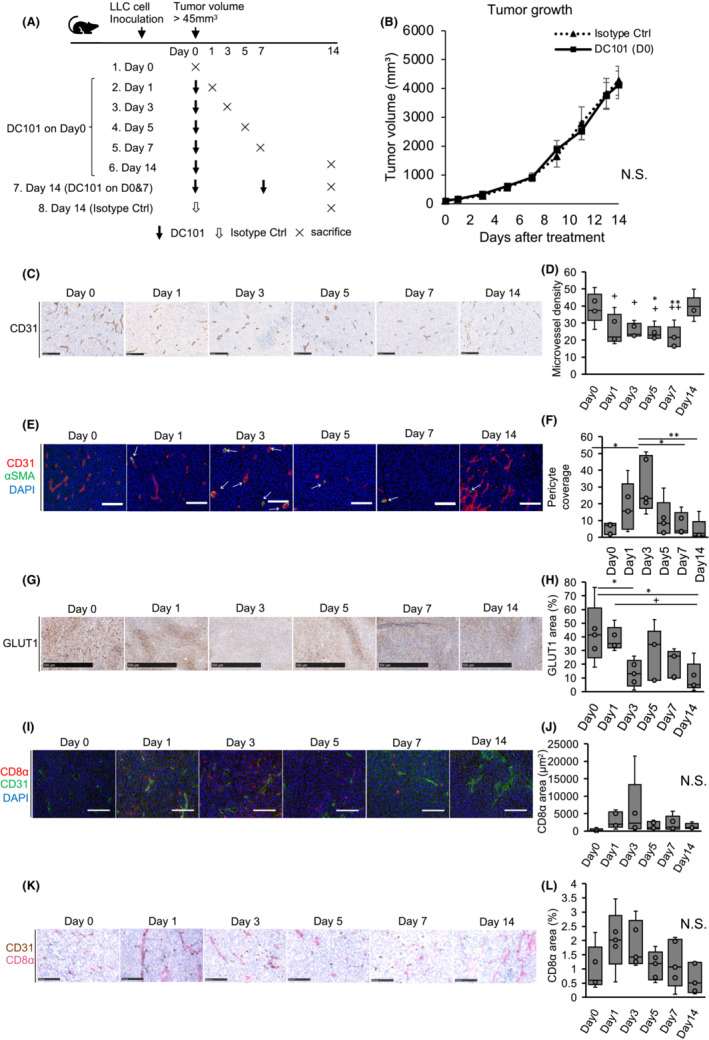
Evaluation of vascular normalization timing using an angiogenic inhibitor. (A) Experimental DC101 (anti‐vascular endothelial growth factor receptor 2 antibody) treatment design and evaluation of vascular normalization timing by histological analyses. (B) Growth curves of Lewis lung carcinoma tumors in the isotype control (ctrl) (Group 8) and DC101 single treatment groups (Group 2‐6) measured every 2 days. N.S. = not significant. Data are mean ± standard error of the mean. (C) Representative images of CD31+ blood vessels in groups. Scale bar = 100 μm. (D) Quantification of microvessel density (MVD). Density decreased until Day 7. *p < 0.05, **p < 0.01 (vs. Day 0), +*p* < 0.05, ++*p* < 0.01 (vs. Day 14) by Tukey–Kramer test. (E) Representative images of CD31 (red), ɑ‐SMA (green), and DAPI staining (blue) in groups. A white arrow shows pericyte‐covered blood vessels. Scale bar = 100 μm. (F) Quantification of the rate of pericyte‐covered blood vessels. Pericyte coverage significantly increased on Day 3. *p < 0.05, **p < 0.01 by Tukey–Kramer test. (G) Tumor hypoxia was measured using GLUT1 expression. Scale bar = 500 μm. (H) Quantification of GLUT1 staining areas. Tumor hypoxia significantly decreased on Day 3. *p < 0.05 (vs. Day 0), +*p* < 0.05 (vs. Day 1) by Tukey–Kramer test. (I) Representative images of CD8α (red), CD31 (green), and DAPI staining (blue) in groups. Scale bar = 100 μm. (J) Quantification of 5 randomly selected CD8α staining areas. CD8+ cells tended to increase on Day 3. N.S. = not significant. *p* = 0.287 (one‐way ANOVA). (K) Visualization of CD8+ cells in vascular hotspots in groups. CD8α (brown) and CD31 immunostaining (red). Scale bar = 100 μm. (L) Quantification of CD8α staining areas in vascular hotspots. N.S. = not significant. *p* = 0.0869 (one‐way ANOVA).

First, vascular normalization timing in subcutaneously inoculated LLC tumors was addressed by harvesting tumor tissues at different times after DC101 treatment in order to find the timing of AI pre‐administration for the subsequent combination treatment with ICI. DC101, a widely used murine VEGFR2 monoclonal antibody,^43^, ^44^, ^45^ was used as an antiangiogenic when analyzing the effect of an ICI in an immunocompetent murine tumor model. After mice were divided into eight groups (*n* = 5/group), LLC cells were subcutaneously inoculated into the right flank of mice. When the tumor volume exceeded 45 mm^3^. Group 1 (control) was sacrificed (Day 0), while the remaining groups, 2, 3, 4, 5, 6, and 7, were administered DC101 at 800 μg/mouse. Groups 2, 3, 4, 5, and 6 mice were sacrificed after 1, 3, 5, 7, and 14 post‐treatment days, respectively (Figure 1A). Mice receiving a DC101 single dose did not exhibit significant tumor volume reduction when compared to isotope control mice in Group 8 (Figure 1B). MVD was noted to gradually decrease until Day 7 in a time‐dependent manner. On Day 14, MVD became larger again (Figure 1C,D). The DC101 twice‐treated Group 7 had a smaller MVD than the single‐treated group at Day 14 (comparable to the single‐treated group on Day 7 (Figure S1)).

Next, we analyzed vessel maturity using pericyte coverage, which is a blood vessel maturation phenotype; α‐SMA‐stained regions were all perivascular and were considered pericytes. Pericyte coverage was calculated as the ratio of α‐SMA‐positive to CD31‐positive areas. Day 3 tumors showed significantly increased pericyte coverage (Figure 1E,F). GLUT1‐positive areas, showing tumor hypoxia, were significantly decreased on Day 3 after single time DC101 treatment (Figure 1G,H), which may have been caused by vessel maturation, with pericyte coverage indicating improved blood perfusion. Next, tumor infiltration by CD8‐positive lymphocytes was analyzed by CD8α staining; CD8‐positive T cells tended to increase on Day 3 after single time DC101 treatment (Figure 1I,J). Tumor‐infiltrating lymphocytes at vascular hotspots had a similar tendency to increase from Days 1 to 3 (Figure 1K,L). Thus, blood vessels in LLC tumors were mostly normalized at Day 3 after DC101 administration. CD11b and Gr‐1‐positive MDSCs tended to increase at Day 3, presumably suggesting normalized blood vessels may have increased infiltration of immune‐suppressing cells (Figure S2A,B).

### 3.2 Pre‐treatment with an AI improves ICI effects

**FIGURE 2:**
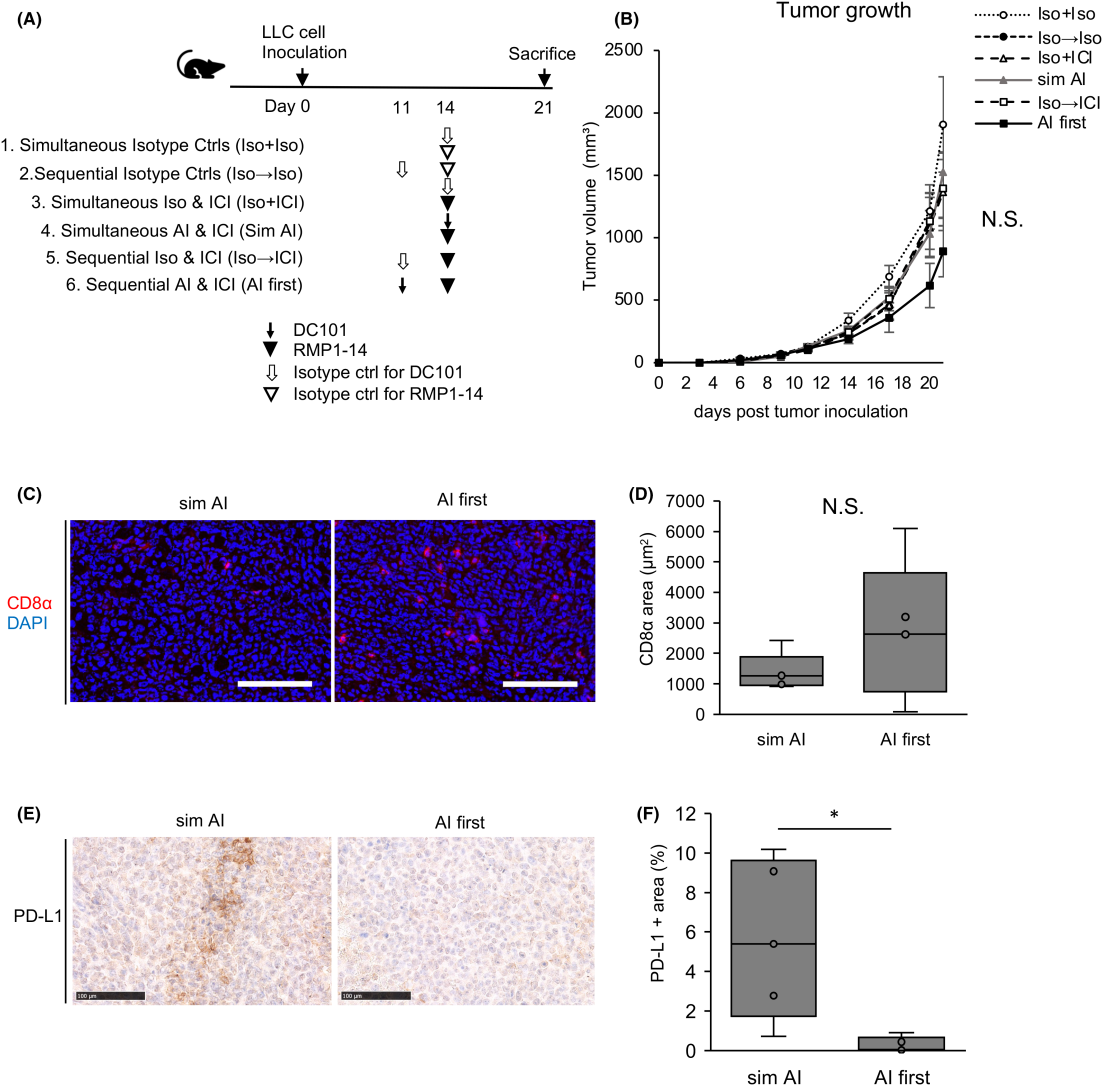
Effects of an immune checkpoint inhibitor after initial antiangiogenic therapy. (A) Experimental DC101 (anti‐vascular endothelial growth factor receptor 2 antibody) and RMP1‐14 (anti‐PD‐1 antibody) treatment design. (B) Tumor growth curves in groups. N.S. = not significant (Tukey–Kramer test). Data are the mean ± standard error of the mean. (C) Representative images of CD8α immunostaining (red) and DAPI staining in sim AI and AI first groups. Scale bar = 100 μm. (D) Quantification of CD8α staining areas. CD8+ cells tended to infiltrate more in the AI first group. N.S. = not significant (Student's *t*‐test). (E) Representative PD‐L1 immunostaining images in sim AI and AI first groups. Scale bar = 100 μm. (F) Quantification of PD‐L1 staining areas. PD‐L1‐positive areas were significantly decreased in the AI first group. *p < 0.05 by Student's *t*‐test.

Next, Venus‐Rluc‐transfected LLC cells were subcutaneously injected into mice; the therapeutic efficacy was then compared between a combination treatment of simultaneous DC101 and RMP1‐14 (anti‐PD‐1 monoclonal antibody) and an initial treatment with DC101, followed by RMP1‐14 within the vascular normalization timing period. Mice were divided into six groups: simultaneous isotype control (Group 1), sequential isotype control (Group 2), simultaneous isotype control for DC101 and RMP1‐14 (Group 3), simultaneous DC101 and RMP1‐14 (Group 4, sim AI), sequential isotype control for DC101 and RMP1‐14 (Group 5), and sequential DC101 and RMP1‐14 (Group 6, AI first) (Figure 2A). The tumor volume of AI first group (Group 6) tended to be smaller than the other groups (Figure 2B). CD8‐positive lymphocytes were more infiltrated in the AI first group than the sim AI group (Group 4) (Figure 2C,D). PD‐L1 immunostaining showed PD‐L1‐positive areas were significantly reduced in the AI first group tumor, suggesting tumor shrinkage was attributed to the antitumoral effects of the anti‐PD‐1 antibody in this group (Figure 2E,F). No metastatic lesions were detected in the lungs of AI‐ and ICI‐treated groups (Groups 4 and 6) by ex vivo bioluminescence imaging (Figure S3).

### 3.3 A pre‐AI strategy improves the antitumoral effects of ICI and paclitaxel

**FIGURE 3:**
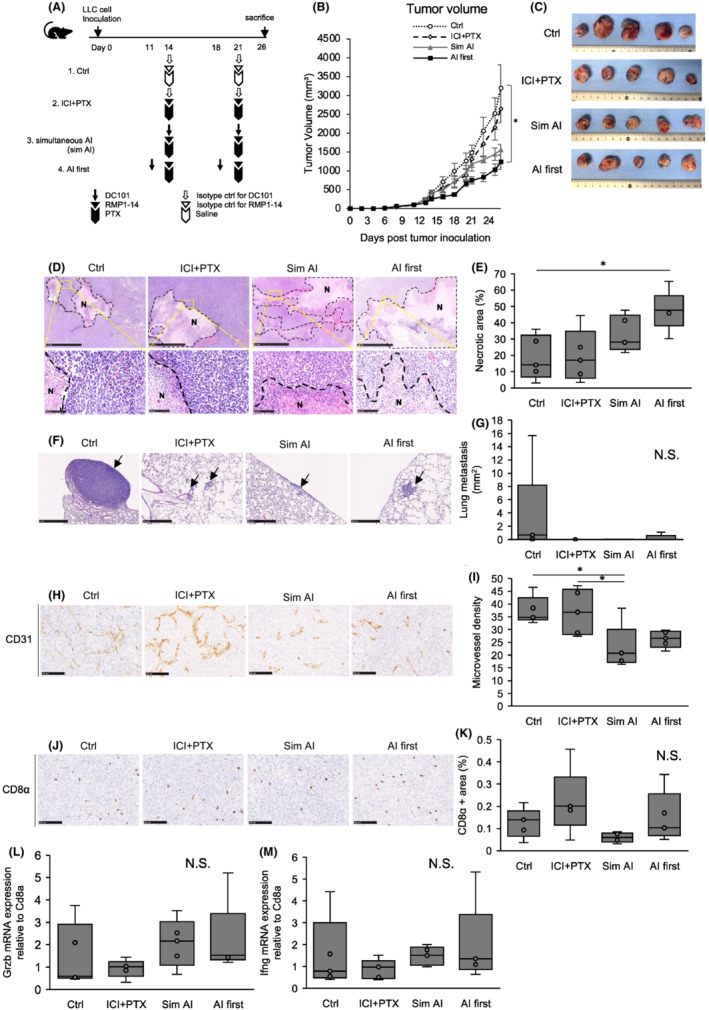
Pre‐angiogenic inhibitor strategy improves the antitumoral effects of an immune checkpoint inhibitor and paclitaxel. (A) Experimental DC101 (anti‐VEGFR2 antibody), RMP1‐14 (anti‐PD‐1 antibody), and paclitaxel treatment design. (B) Tumor growth curves of groups. The AI first group showed significant tumor volume reductions. *p < 0.05 (Tukey–Kramer test). Data are the mean ± standard error of the mean. (C) Macroscopic tumor appearance.(D) Representative images of necrotic areas by hematoxylin–eosin staining in groups. N = necrotic area. Scale bar = lower magnification: 2.5 mm, higher magnification: 100 μm. (E) Quantification of necrotic areas. These areas were significantly larger in the AI first group. *p < 0.05 (Tukey–Kramer test). (F) Representative images of lung metastases in groups. Black arrows = metastatic lesions. Scale bar = 1 mm (Ctrl) and 500 μm (ICI + PTX, Sim AI and AI first). (G) Quantification of lung metastatic areas. Lung metastases tended to decrease in treated groups. N.S. = not significant, *p* = 0.348 (one‐way ANOVA). (H) Representative images of CD31+ stained blood vessels in groups. Scale bar = 100 μm. (I) Quantification of microvessel density (MVD). The sim AI group had a significantly lower MVD. *p < 0.05 (Tukey–Kramer test). (J) Representative images of CD8α immunostaining in groups. Scale bar = 100 μm. (K) Quantification of CD8α staining areas. CD8+ cells in the AI first group tended to increase when compared to the sim AI group. N.S. = not significant, *p* = 0.133 (one‐way ANOVA). (L) Granzyme B mRNA expression in CD8+ T cells in AI‐treated groups tended to be higher than in other groups. N.S. = not significant, *p* = 0.383 (one‐way ANOVA). (M) Interferon‐γ mRNA expression in CD8+ T cells in AI‐treated groups tended to be higher than in other groups. N.S. = not significant, *p* = 0.632 (one‐way ANOVA).

To reflect lung cancer clinical practice where AIs are regularly used in combination with other cytotoxic antineoplastic agents, LLC tumor‐bearing mice were treated with DC101, RMP1‐14, and paclitaxel. The control (Ctrl) group mice received saline and isotype controls. The ICI + PTX group mice were treated with RMP1‐14 and paclitaxel. The sim AI group mice were simultaneously treated with DC101, RMP1‐14, and paclitaxel. The AI first group mice were pre‐treated 3 days prior with DC101 followed by RMP1‐14 and paclitaxel (AI first) (Figure 3A). When compared to the control group, only the AI first group showed statistically significant reduced tumor volume (Figure 3B,C). Hematoxylin–eosin staining revealed this group showed significantly increased necrotic areas (Figure 3D,E). This suggested that ICI and paclitaxel administration when the tumor blood vessels are normalized improved anti‐tumor effects by improving intratumoral drug delivery and immune cell infiltration. Lung metastases decreased in treated groups when compared to the control group (Figure 3F,G). MVD in the sim AI group was significantly lower than in control and ICI + PTX groups and tended to be lower than the AI first group, suggesting that antiangiogenic effects in the AI first group had already been abrogated by the time tumors were harvested (Figure 3H,I). Thus, increased tumor necrosis could be attributed to the therapeutic effects of ICI and PTX, rather than the antiangiogenic effects of DC101. CD8‐positive cytotoxic T lymphocytes tended to increase in the AI first group (Figure 3J,K), and granzyme B and IFN‐γ expression in whole tumors tended to increase in groups treated with DC101 (Figure 3L,M). The granzyme B‐positive CD8‐positive T cells tended to increase in the AI first group compared with the sim AI group (Figure S4A,B). These data suggested that CD8‐positive T cells retained cytotoxicity via tumor blood vessel normalization and accompanying favorable changes in the tumor microenvironment.

### 3.4 A pre‐AI strategy may be generalized in other cancer types

**FIGURE 4:**
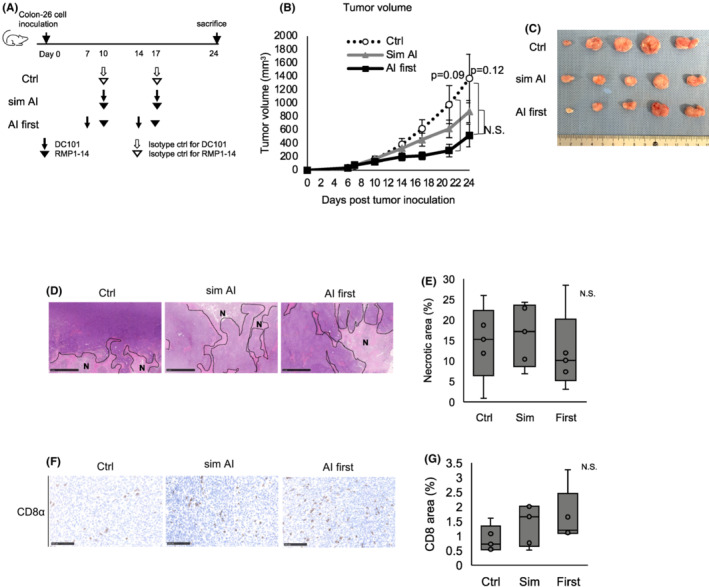
Pre‐angiogenic inhibitor strategy tends to improve the ICI antitumoral effects in colon cancer model. (A) Experimental DC101 and RMP1‐14 treatment design. (B) Tumor growth curves. The AI group tended to inhibit tumor growth more. Data are presented as the mean ± standard error. Tukey–Kramer test. N.S. = not significant. (C) Macroscopic tumor appearance. (D) Representative necrotic area images by hematoxylin–eosin staining in groups. N = necrotic area. Scale bar = 1 mm. (E) Quantification of necrotic areas. N.S. = not significant. *p* = 0.76 (one‐way ANOVA). (F) CD8‐positive cell representative images in tumors. Scale bar = 100 μm. (G) Quantification of CD8‐positive cell infiltration in the whole tumor section. The AI first group tended to have more CD8‐positive cell infiltration. N.S. = not significant. *p* = 0.27 (one‐way ANOVA).

Colon‐26 tumor‐bearing mice were treated with isotype controls (ctrl), simultaneous AI and ICI (sim AI) or AI pre‐administration followed by ICI 3 days later (AI first) (Figure 4A) to investigate whether the aforementioned findings on the superiority of pre‐AI strategy can be extended to other cancer types. Tumor growth tended be most suppressed (Figure 4B,C) in the AI first group. There was no difference in the necrotic area ratio between the groups (Figure 4D,E). The tumor‐infiltrating lymphocytes tended to increase in the AI first group (Figure 4F,G). These data suggested AI pre‐administration may increase anti‐tumor effects mediated by increased cytotoxic lymphocyte infiltration.

## 4 DISCUSSION

**FIGURE 5:**
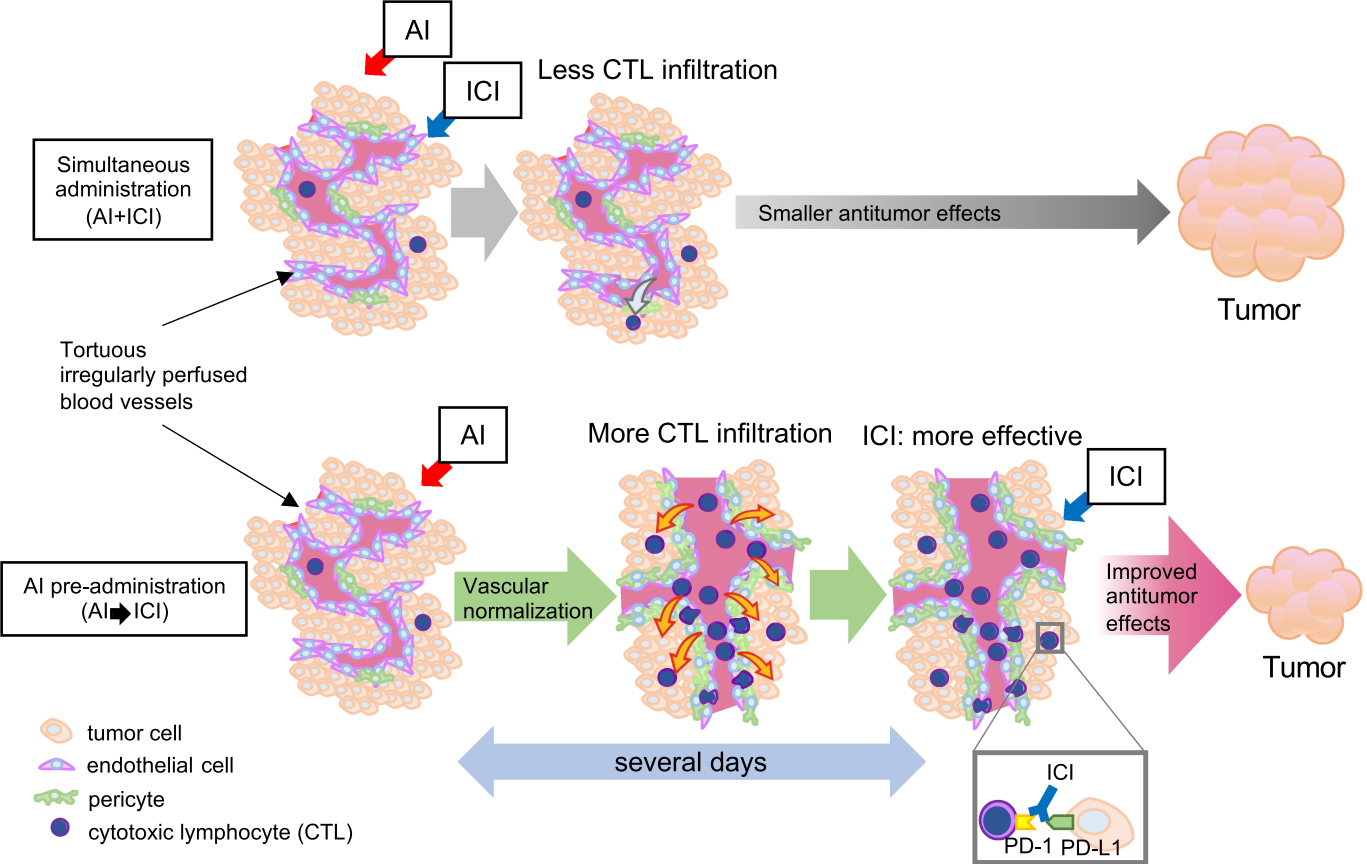
Pre‐angiogenesis inhibitor strategy improves immune checkpoint inhibitor effects by improving CD8 infiltration and drug delivery. The current clinical protocol of administering AI and ICI on the same day results in ICI acting when vascular normalization is insufficient to mobilize CTL in the tumor tissue (upper panel). By contrast, the anti‐tumor effect is enhanced when AI is administered several days before ICI, as ICI is given after vascular normalization and the number of immune cells infiltrating into the tissue has increased (lower panel).

As per our findings, we were able to demonstrate that pre‐administration with AI improved immunotherapy efficacy in lung cancer when compared to concomitant administration (Figure 5), which is the currently approved treatment schedule. We showed that vascular normalization prior to immunotherapy enhanced antitumoral effects.

ICIs have drastically changed the clinical treatment of different cancer types. However, when used as a monotherapy, clinical study response rates can be as low as 20%,^46^, ^47^ with many patients demonstrating no substantial therapeutic benefits. It is clear that intratumoral lymphocyte infiltration is essential for effective immunotherapy. The therapeutic efficacy of PD‐1/PD‐L1 blockade correlates well with intratumoral CD8‐positive cells.^48^, ^49^ However, due to abnormal and dysfunctional blood vessels, tumor tissues are prone to reduced infiltration by effector immune cells^50^; they are more hypoxic and acidic,^51^ which could lead to increased VEGF expression and immune checkpoint molecule expression in T cells.^52^, ^53^ These tumor microenvironment characteristics inhibit T‐cell cytotoxic activity; therefore, vascular normalization by AIs is important for effective antitumoral immune responses.

Currently, in many cancers, AIs are used for vascular normalization effects in combination with other antineoplastic drugs and have largely exhibited improved therapeutic effects.^54^, ^55^ In non‐small cell lung cancer, bevacizumab, an anti‐human VEGF monoclonal antibody, first improved patient survival and response rates when combined with carboplatin and paclitaxel^54^ and later improved progression‐free survival when combined with carboplatin, paclitaxel, and atezolizumab in high PD‐L1‐expressed tumors.^55^ In previously treated cancer patients, ramucirumab, an anti‐human VEGFR2 monoclonal antibody, improved progression‐free survival when combined with docetaxel.^56^ However, in contrast, although ICIs are added to conventional cytotoxic antineoplastic agents and AIs to improve the therapeutic efficacy, their additive effects remain to be fully exploited. Thus, many patients have not benefited from combination therapy.^33^

Previous studies reported that vascular normalization effects by AIs were transient, and several days were required from drug administration to generate normalization effects,^57^, ^58^, ^59^ consistent with this study. Thus, for currently approved therapies, combination drugs are administered when tumor blood vessels are not sufficiently normalized. Indeed, it was reported that AI administration prior to anticancer drug administration increased antitumoral effects in murine experimental models, for example, an initial apatinib dose (VEGFR2 tyrosine kinase inhibitor) was more effective than concomitant administration with pemetrexed.^59^ A mathematical model study also demonstrated improved antitumoral effects when bevacizumab was administered prior to cisplatin and pemetrexed.^38^ Therefore, vascular normalization facilitated intratumoral drug delivery. Nevertheless, the effects of initial vascular normalization in lung cancer immunotherapy using ICIs have not been addressed before. To the best of our knowledge, this study is the first to show that VEGFR2 antibody pre‐administration was more effective than concomitant administration with an ICI and a cytotoxic antineoplastic drug in an immunocompetent mouse model, focusing on the effects of vascular normalization. This model enabled us to perform histological analysis on tumor vasculature and immune cell infiltration at different timepoints after drug treatment which is a surrogate for repetitive biopsies that is impossible to perform on cancer patients in real clinical settings. Our results suggested that improved antitumoral responses were attributed to improved CD8‐positive cell infiltration. However, other immune modulating effects in the tumor microenvironment, such as the effects on immune checkpoint protein expression in T cells and protumoral immunosuppressive cell infiltration, require further investigation. The CD8‐positive cell number tended to increase, though not significantly in this study (Figures 2 and 3). Considering the data in Figure 1, CD8‐positive T cells increased on Day 3 after AI administration, but in the evaluation on 5–8 days after AI administration in Figures 2 and 3, the vascular normalization effect of AI may have expired and the difference in CD8‐positive T‐cell mobilization was not detected well. Other study limitations include the use of LLC tumor cells, which are a well‐established syngeneic murine lung cancer cell line. The cells are known to be poorly immunogenic^60^ and did not exhibit tumor growth inhibition in the AI and ICI combination model. By investigating the pre‐administration AI effects on colon‐26 tumor, the study was able to suggest that improved therapeutic effects can be observed on another tumor model. The effects of initial vascular normalization in other tumor models should be additionally examined. Another limitation was that while we used a subcutaneous model, vasculature and immune cell infiltration effects may be different in an orthotopic or spontaneous tumor model.^61^, ^62^ Moreover, the kinetics of anti‐human VEGF/VEGFR2 monoclonal antibodies in clinical use may be different from the drugs which were used in our mouse models. These factors must be considered when translating our results to clinical settings.

To implement our strategy in clinical practice, vascular normalization timing must be monitored. However, monitoring methods have not yet been established without repeated tumor tissue biopsies following antiangiogenic treatments, which are unacceptable for cancer patients. Although previous preclinical studies proposed serological markers or imaging technologies,^58^, ^63^ no vascular normalization detection methods are clinically available and thus require development. Finally, we provided preclinical insights for future clinical studies to verify the superiority of an initial vascular normalization strategy to improve patient survival.

## AUTHOR CONTRIBUTIONS

**Mineyoshi Sato:** Data curation (lead); formal analysis (lead); investigation (lead); methodology (equal); visualization (equal); writing – original draft (lead). **Nako Maishi:** Funding acquisition (equal); methodology (lead); project administration (equal); writing – original draft (supporting). **Yasuhiro Hida:** Funding acquisition (equal); methodology (supporting). **Aya Yanagawa‐Matsuda:** Funding acquisition (equal); methodology (supporting). **Mohammad Towfik Alam:** Resources (supporting). **Jun Sakakibara‐Konishi:** Methodology (supporting). **Jin‐Min Nam:** Resources (supporting). **Yasuhito Onodera:** Resources (supporting). **Satoshi Konno:** Methodology (supporting). **Kyoko Hida:** Conceptualization (lead); funding acquisition (equal); methodology (supporting); project administration (equal); supervision (lead); writing – original draft (supporting).

## FUNDING INFORMATION

This research was supported by the JSPS Grants‐in‐Aid for Scientific Research to NM (JP21K10107), AYM (JP20K09900), YH (JP21H03019), and KH (JP21H04840), Grants from Japan Agency for Medical Research and Development (AMED) to KH (JP20ck0106406h0003).

## CONFLICT OF INTEREST STATEMENT

The authors have no conflict of interest.

## ETHICS STATEMENT

Animal studies: All animal procedures were performed in accordance with institutional guidelines and approved by the Institutional Animal Care and Use Committee of Hokkaido University.

## Supporting information


**Figure S1.** Microvessel density of Group 6 (DC101 on Day 0), Group 7 (DC101 on Day 0 & 7), and Group 8 (Isotype Ctrl on Day 0).

**Figure S2.** Analysis of myeloid‐derived suppressor cells (MDSCs) in LLC tumors in DC101 monotherapy model. (A) CD11b + Gr‐1 + MDSCs representative images in the tumor. White arrows = MDSCs. Scale bar = 100 μm. (B). Quantification of MDSCs. MDSCs were not suppressed by single‐dose DC101. N.S.,not significant; *p* = 0.096 (one‐way ANOVA).

**Figure S3.** Ex vivo imaging of lung metastasis in each group. Tumor cell luminescence in the lungs was detected using IVIS Spectrum.

**Figure S4.** Granzyme B ‐positive CD8 cell analysis in AI, ICI, and paclitaxel model.
(A) Granzyme B and CD8‐positive cells representative images in the tumor. White arrows = granzyme B‐positive CD8‐positive cells. Scale bar = 100 μm.
(B) Quantification of granzyme B‐positive CD8‐positive cells ratio among all CD8‐positive cells. N.S.,not significant; *p* = 0.62 (one‐way ANOVA).

## Data Availability

The data that support the findings of this study are available from the corresponding author upon reasonable request.
