# Supplementary material for: Angiogenic inhibitor pre‐administration improves the therapeutic effects of immunotherapy
Source: Cancer Med. 2023 Feb 19;12(8):9760–73. doi: 10.1002/cam4.5696 (PMC10166916; doi:10.1002/cam4.5696)

Fig. S1. Microvessel density of Group 6 (DC101 on Day 0), Group 7 (DC101 on Day 0 & 7), and Group 8 (Isotype Ctrl on Day 0). * $p < 0.05$ (Tukey-Kramer test).

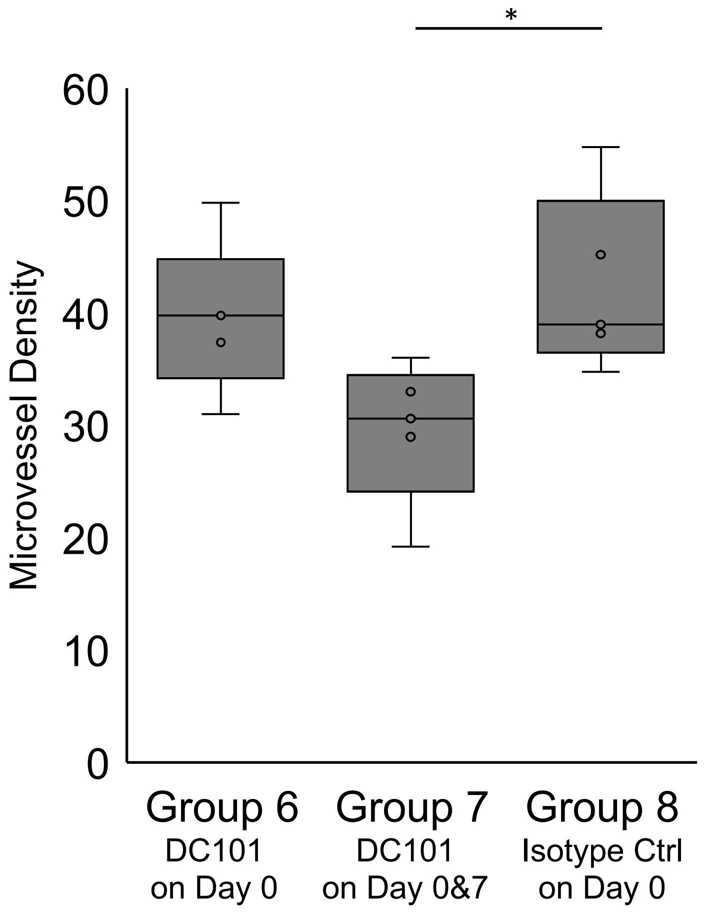

Supplement: Supplementary file 1 — Figure S1. Microvessel density of Group 6 (DC101 on Day 0), Group 7 (DC101 on Day 0 & 7), and Group 8 (Isotype Ctrl on Day 0). [file CAM4-12-9760-s002.pdf]
