# Supplementary material for: Angiogenic inhibitor pre‐administration improves the therapeutic effects of immunotherapy
Source: Cancer Med. 2023 Feb 19;12(8):9760–73. doi: 10.1002/cam4.5696 (PMC10166916; doi:10.1002/cam4.5696)

Fig. S2. Analysis of myeloid-derived suppressor cells (MDSCs) in LLC tumors in DC101 monotherapy model.

A. CD11b+ Gr-1+ MDSCs representative images in the tumor. White arrows = MDSCs. Scale bar = 100 µm.

B. Quantification of MDSCs. MDSCs were not suppressed by single-dose DC101. N.S. = not significant, p = 0.096 (one-way ANOVA).

A.

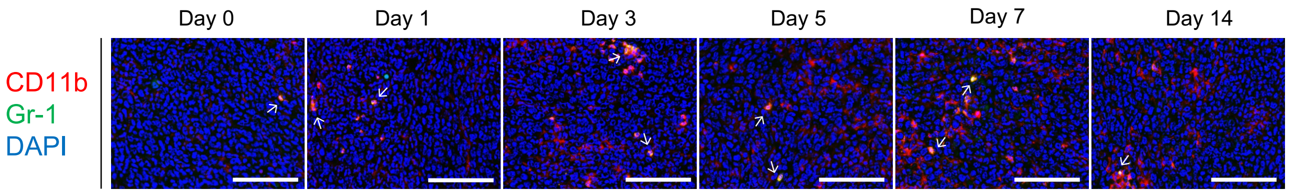

B.

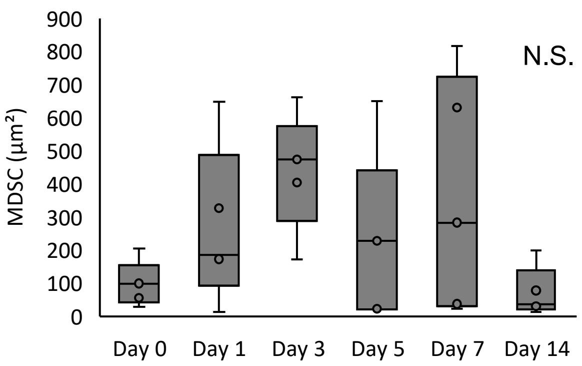

Supplement: Supplementary file 2 — Figure S2. Analysis of myeloid‐derived suppressor cells (MDSCs) in LLC tumors in DC101 monotherapy model. (A) CD11b + Gr‐1 + MDSCs representative images in the tumor. White arrows = MDSCs. Scale bar = 100 μm. (B). Quantification of MDSCs. MDSCs were not suppressed by single‐dose DC101. N.S.,not significant; p = 0.096 (one‐way ANOVA). [file CAM4-12-9760-s004.pdf]
