# Supplementary material for: Angiogenic inhibitor pre‐administration improves the therapeutic effects of immunotherapy
Source: Cancer Med. 2023 Feb 19;12(8):9760–73. doi: 10.1002/cam4.5696 (PMC10166916; doi:10.1002/cam4.5696)

Fig. S3. Ex vivo imaging of lung metastasis in each group. Tumor cell luminescence in the lungs was detected using IVIS Spectrum.

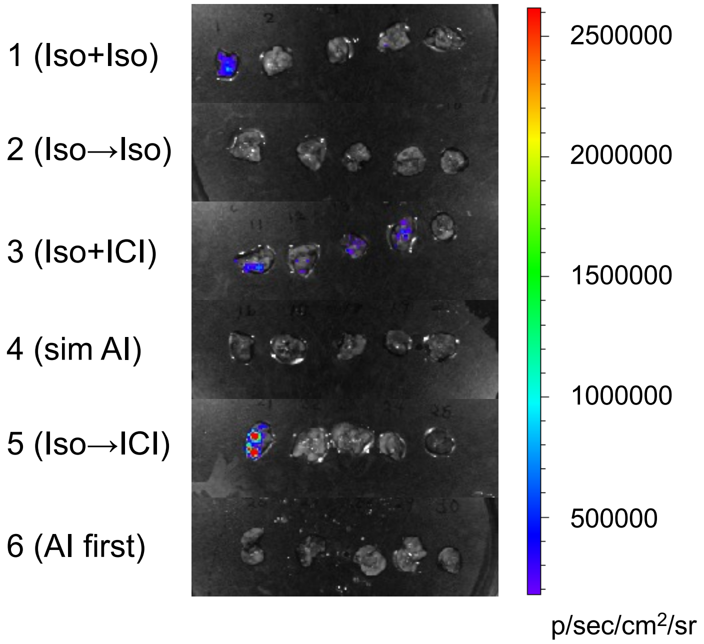

Supplement: Supplementary file 3 — Figure S3. Ex vivo imaging of lung metastasis in each group. Tumor cell luminescence in the lungs was detected using IVIS Spectrum. [file CAM4-12-9760-s001.pdf]
