# Supplementary material for: Angiogenic inhibitor pre‐administration improves the therapeutic effects of immunotherapy
Source: Cancer Med. 2023 Feb 19;12(8):9760–73. doi: 10.1002/cam4.5696 (PMC10166916; doi:10.1002/cam4.5696)

Fig. S4. Granzyme B -positive CD8 cell analysis in AI, ICI and paclitaxel model.
A. Granzyme B and CD8-positive cell representative images in the tumor.
White arrows = granzyme B-positive CD8-positive cells. Scale bar = 100 μm.
B. Quantification of granzyme B-positive CD8-positive cells ratio among all CD8-positive cells.
N.S. = not significant, p = 0.62 (one-way ANOVA).

A.

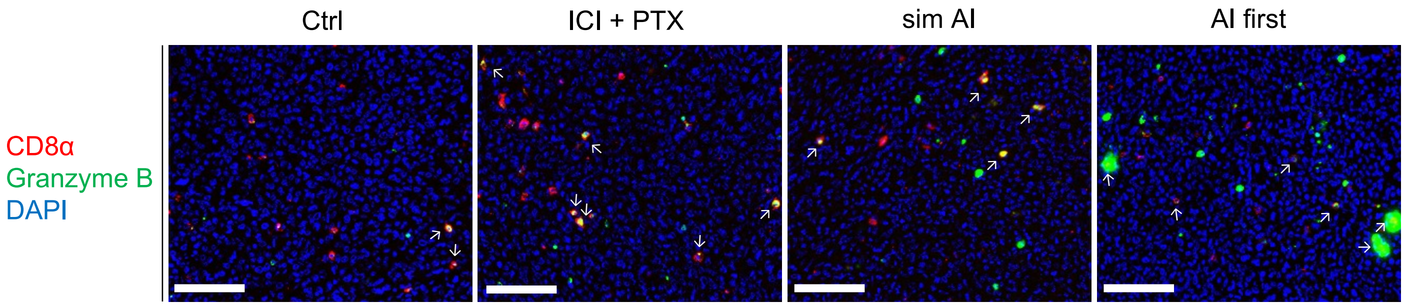

B.

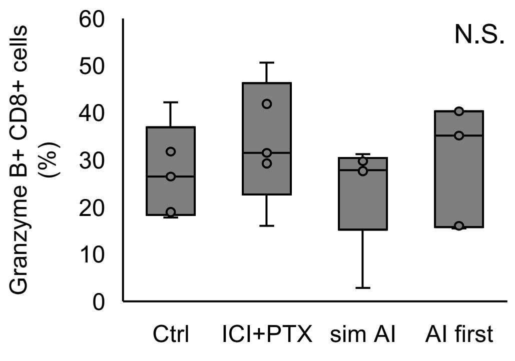

Supplement: Supplementary file 4 — Figure S4. Granzyme B ‐positive CD8 cell analysis in AI, ICI, and paclitaxel model. (A) Granzyme B and CD8‐positive cells representative images in the tumor. White arrows = granzyme B‐positive CD8‐positive cells. Scale bar = 100 μm. (B) Quantification of granzyme B‐positive CD8‐positive cells ratio among all CD8‐positive cells. N.S.,not significant; p = 0.62 (one‐way ANOVA). [file CAM4-12-9760-s003.pdf]
